# A Reconstruction Method of Blood Flow Velocity in Left Ventricle Using Color Flow Ultrasound

**DOI:** 10.1155/2015/108274

**Published:** 2015-05-19

**Authors:** Jaeseong Jang, Chi Young Ahn, Kiwan Jeon, Jung Heo, DongHak Lee, Chulmin Joo, Jung-il Choi, Jin Keun Seo

**Affiliations:** ^1^Department of Computational Science and Engineering, Yonsei University, Seoul 120-749, Republic of Korea; ^2^Division of Computational Mathematics, National Institute for Mathematical Sciences, Daejeon 305-811, Republic of Korea; ^3^School of Mechanical Engineering, Yonsei University, Seoul 120-749, Republic of Korea

## Abstract

Vortex flow imaging is a relatively new medical imaging method for the dynamic visualization of intracardiac blood flow, a potentially useful index of cardiac dysfunction. A reconstruction method is proposed here to quantify the distribution of blood flow velocity fields inside the left ventricle from color flow images compiled from ultrasound measurements. In this paper, a 2D incompressible Navier-Stokes equation with a mass source term is proposed to utilize the measurable color flow ultrasound data in a plane along with the moving boundary condition. The proposed model reflects out-of-plane blood flows on the imaging plane through the mass source term. The boundary conditions to solve the system of equations are derived from the dimensions of the ventricle extracted from 2D echocardiography data. The performance of the proposed method is evaluated numerically using synthetic flow data acquired from simulating left ventricle flows. The numerical simulations show the feasibility and potential usefulness of the proposed method of reconstructing the intracardiac flow fields. Of particular note is the finding that the mass source term in the proposed model improves the reconstruction performance.

## 1. Introduction

Vortex flow imaging has recently attracted much attention in the field of clinical cardiac assessment owing to reports of its feasibility for analyzing intraventricular vortex flows [1–3]. The vorticity of intraventricular blood flow describes a rotational flow pattern that offers possible clinical indices of cardiac functions such as sphericity, vortex depth, vortex length, and vortex pulsation correlation.

There are several methods to compute and visualize the velocity fields of blood flow inside the left ventricle (LV), with echo particle image velocimetry (E-PIV) being representative of the commonly used noninvasive methods [4]. It tracks the speckle patterns of blood flow to estimate blood motion within the imaging plane. Although it is generally unable to measure out-of-plane particle motion from 2D echocardiography data (called B-mode images), a recent study extending E-PIV to 3D volume data demonstrated the possibility of out-of-plane assessment [5]. However, E-PIV is not completely noninvasive because it requires the intravenous injection of a contrast agent to obtain images suitable for the speckle-tracking algorithm.

To develop less invasive techniques, methods to reconstruct blood flows from color flow images (also called C-mode images, color Doppler images, color Doppler data, or Doppler echocardiography) have been proposed. The color flow images reflect the projected velocity components in the direction of ultrasound beam propagation [6]. To compute the flow velocity from color flow images, Garcia et al. [7] assumed a 2D divergence-free condition on the velocity fields; they decomposed each 2D velocity vector into a radial component obtained from the color flow data and an unknown angular component, which was computed using their assumption of the 2D flow. Ohtsuki and Tanaka [8] also assumed 2D flows and recovered the 2D velocity fields from the color Doppler data using the concepts of stream function and streamline in 2D fluid flow. However, the assumption of a 2D divergence-free condition is an oversimplification that ignores out-of-plane flows.

Arigovindan et al. [9] proposed a velocity reconstruction method using color Doppler data acquired from beams in two different directions. To cope with the nonuniformly sampled data of multiple imaging planes, they used 2D B-spline on each of the velocity components to be estimated, and the unknown coefficients of the 2D B-spline were calculated from the measured color Doppler data using least squares. Similar to the 2D reconstruction, Gomez et al. [10] recovered 3D velocity fields from multiple registered color Doppler images using 3D B-spline and least squares. The registration of multiple imaging planes for the above two methods remains very challenging in a practical environment.

Recently, a new imaging modality (Doppler vortography) based on 2D color Doppler data was introduced by Mehregan et al. [11], who assumed that a vortex flow pattern has axisymmetric features in the neighborhood of its center. Their method employs a simple kernel filter designed to find the positions of axisymmetry in the 2D color Doppler images. The vortex flow was recovered using a color Doppler-variable vorticity function that directly computes vorticities from color Doppler values. However, the assumption of axisymmetry does not reflect detailed flow patterns, and it may lead to inaccurate vortex positions and vorticity values in patients with severe dysfunction where axisymmetry cannot be assumed at all.

In this paper, we propose a 2D Navier-Stokes model to reconstruct intraventricular flows using color flow images and LV boundaries extracted from echocardiography data. Although the use of the full 2D Navier-Stokes equations in this setting has already been proposed and evaluated for 2D flow field regularization [12], the originality of this work is the inclusion of a source-term to deal with the out-of-plane flow component. The proposed model considers both in-plane and out-of-plane blood flows for an imaging plane in apical long-axis three-chamber (A3CH) view. Particular attention is given to the appearance and disappearance of the out-of-plane components in the imaging plane, which is modeled as a mass source term of a source-sink distribution. Blood flows in the imaging domain are reconstructed through solving a system of equations, which include a 2D incompressible Navier-Stokes equation for the mass source term and the color flow data measurement equation describing the projected velocity component for the color flow data. The boundary conditions required to solve the system of equations are given by the LV borders extracted from echocardiography data.

The performance of the proposed method is evaluated numerically using synthetic flow data with LV motion. The proposed method is shown to be feasible and potentially valuable for reconstructing intracardiac flow fields.

## 2. Materials and Methods

Commonly used ultrasound systems can provide not only 2D echo images but also color flow images, which represent the scanline directional components of the velocity fields using the phase-shift estimated by a standard autocorrelation algorithm [13]. Our flow reconstruction method is to reconstruct the intraventricular flows using color flow images and the LV boundaries extracted from the echo images. In this section, we describe the overall outline of our flow reconstruction method using those ultrasound measurements based on two assumptions as follows:

- the time difference between sequential color flow imaging frames is very small;
- the echo and color flow images are acquired simultaneously and separately for the entire heart cycle.

### 2.1. Mathematical Model on 2D Imaging Plane

**Figure 1:**
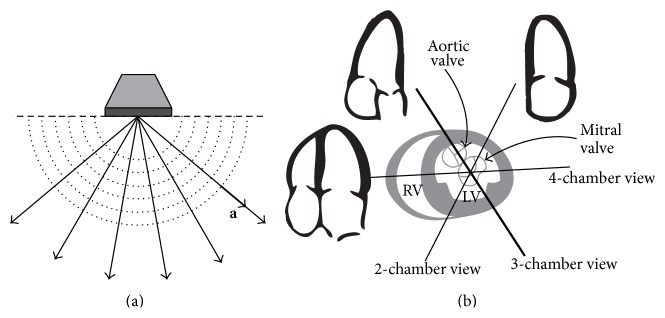
A sector scanning probe and apical long-axis views. The vector **a** in (a) means ultrasound beam propagation directions in sector-type scanning for cardiac application. (b) shows three (4-chamber, 3-chamber, and 2-chamber) apical imaging views for the cardiac scanning. They have the relationship of rotation by approximately 60 degrees toward each other. The imaging plane of the apical long-axis 3-chamber view passes through both of mitral and aortic valves.

We focus on the dominant vortex flow appearing in the A3CH view, which passes through the apex and the mitral and aortic valves as shown in Figure 1(b), and mathematical model for blood flows inside the LV on the imaging plane of the A3CH view.

**Figure 2:**
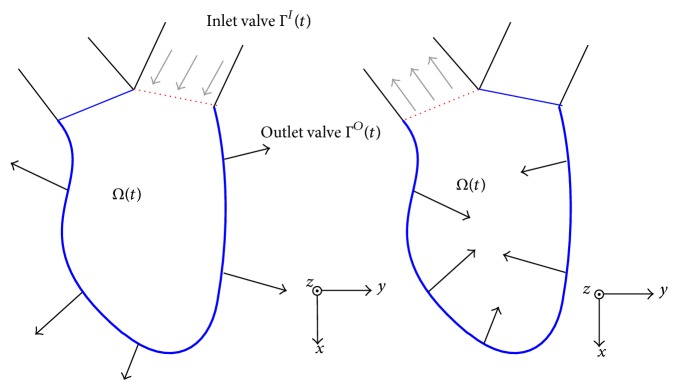
Description on the LV region *Ω*(*t*) in the A3CH view. They show the diastolic and systolic motion of  *Ω*(*t*), respectively. Here, Γ^*I*^(*t*) and Γ^*O*^(*t*) refer to the mitral and aortic valves (as inlet and outlet valves), respectively.

Let *D* be a 2D imaging domain and *Ω*(*t*) the cross-section of the LV region in the A3CH view so that they satisfy *Ω*(*t*)⊆*D*⊆*ℝ* ^2^. Color flow data are practically measured in the imaging plane *D*.  Figure 1(a) describes the scanline directional vectors **a** : = (*a*_1_, *a*_2_) for the 2D imaging plane *D* as an example of sector scanning. As shown in Figure 2, Γ^*I*^(*t*) and Γ^*O*^(*t*) denote the mitral and aortic valves, respectively. The parameter *t* denotes the LV region and valves as time-varying boundaries. The superscripts *I* and *O* of Γ^*I*^(*t*) and Γ^*O*^(*t*) stand for inlet and outlet valves, respectively. The 3D coordinate system takes the *xy*-plane to contain *Ω*(*t*) and the *z*-axis to be normal to this plane. Figure 2 describes a basic LV structure and the diastolic/systolic motion of its wall observed in the A3CH view.

Let *c*(**x**, *t*) be the measured color flow data and (*u*(**x**, *t*), *v*(**x**, *t*)) the velocity fields of flow at the position **x** ∈ *D* and time *t*, respectively. Then the color flow data *c*(**x**, *t*) can be expressed as the inner product of the scanline vector and the velocity vector:

(1) $$\begin{array}{c} c\left(x,t\right)=\left(a_{1}\left(x\right),a_{2}\left(x\right)\right)\cdot \left(u\left(x,t\right),v\left(x,t\right)\right). \end{array}$$

To recover (*u*, *v*) from knowledge of *c* on the imaging plane *D*, we propose the following 2D Navier-Stokes model:

(2) $$\begin{array}{c} \frac{\partial u}{\partial t}+u\frac{\partial u}{\partial x}+v\frac{\partial u}{\partial y}=-\frac{1}{\rho }\frac{\partial p}{\partial x}+\frac{\mu }{\rho }\nabla^{2}u+\frac{\mu }{3\rho^{2}}\frac{\partial s}{\partial x},\\ \frac{\partial v}{\partial t}+u\frac{\partial v}{\partial x}+v\frac{\partial v}{\partial y}=-\frac{1}{\rho }\frac{\partial p}{\partial y}+\frac{\mu }{\rho }\nabla^{2}v+\frac{\mu }{3\rho^{2}}\frac{\partial s}{\partial y},\\ \frac{\partial u}{\partial x}+\frac{\partial v}{\partial y}=\frac{s}{\rho }, \end{array}$$

where *ρ* = 1050 kg/m^3^ and *μ* = 0.00316 Pa·s are the density and viscosity of the blood flow, respectively [14]. This model (2) is equivalent to a 2D incompressible flow having a source-sink distribution *s*(**x**, *t*) [15]. In fact, the 3D blood flows appear or disappear in the imaging plane. Therefore, we reconstruct the 2D velocity fields in the imaging plane by solving (1) and (2) with LV boundary conditions. We impose the following boundary conditions:

(3) $$\begin{array}{c} u=u_{wall}\;on\;\partial \Omega \left(t\right),\\ v=v_{wall}\;on\;\partial \Omega \left(t\right),\\ \frac{\partial p}{\partial n}=0\;on\;\partial \Omega \left(t\right),\\ s=0\;on\;\partial \Omega \left(t\right), \end{array}$$

where *u*_wall_ and *v*_wall_ are velocity components computed by the motion of  ∂*Ω*(*t*) and **n** is the unit outward normal vector to ∂*Ω*(*t*). Here, ∂*Ω*(*t*) is the boundary of  *Ω*(*t*), and its motion can be extracted from echocardiography data as described in the above subsection. Further mathematical explanation of (1) and (2) is given in the Appendix A.

### 2.2. Reconstruction Algorithm

For numerical implementation, we write the system of   (1) and (2) as the following linear second-order system:

(4) a1a200∂∂x∂∂y0−1ρ∂∂t−μρ∇201ρ∂∂x−μ3ρ2∂∂x0∂∂t−μρ∇21ρ∂∂y−μ3ρ2∂∂yuvps  =c0−u∂u∂x−v∂u∂y−u∂v∂x−v∂v∂y.

We discretize the 2D imaging region *Ω* into the mesh grid elements with *M* × *N* nodes and apply the standard finite difference method for the linear equation (4). Then we obtain the discretized linear system of the following form:

(5) a1a200DxDy0−1ρ1−μΔtρL0ΔtρDx−μΔt3ρ2Dx01−μΔtρLΔtρDy−μΔt3ρ2Dyun+1vn+1pn+1sn+1  =cn+10un−ΔtunDxun+vnDyunvn−ΔtunDxvn+vnDyvn,

where *D*_*x*_, *D*_*y*_, and *L* are the *x*-derivative, *y*-derivative, and Laplace operators of finite difference, respectively; the superscript (*n*) denotes the *n*th time-step. Note that the measured data *c* on the right-hand side are the values at the (*n* + 1)th step, not the (*n*)th step.

Let **I** and **O** be the *N* × *N* identity and zero matrices, respectively. Also set **D** and **L** to

(6) $$\begin{array}{c} D=\left[\begin{array}{cccc} 0 & \frac{1}{2h} & & \\ -\frac{1}{2h} & \ddots & \ddots & \\ & \ddots & \ddots & \frac{1}{2h}\\ & & -\frac{1}{2h} & 0 \end{array}\right],\\ L=\frac{1}{h^{2}}\left[\begin{array}{cccc} -4 & 1 & & \\ 1 & \ddots & \ddots & \\ & \ddots & \ddots & 1\\ & & 1 & -4 \end{array}\right], \end{array}$$

and let *𝔸*_1_, *𝔸*_2_, *𝔻*_*x*_, *𝔻*_*y*_, and *𝕃* be the (*M* × *N*)×(*M* × *N*) matrices defined by

(7) $$\begin{array}{c} A_{1}=diag\left({a_{1}}_{\left(1,1\right)},\ldots ,{a_{1}}_{\left(N,M\right)}\right),\\ A_{2}=diag\left({a_{2}}_{\left(1,1\right)},\ldots ,{a_{2}}_{\left(N,M\right)}\right),\\ D_{x}=\left[\begin{array}{ccc} D & & \\ & \ddots & \\ & & D \end{array}\right],\\ D_{y}=\left[\begin{array}{cccc} O & -\frac{1}{2h}I & & \\ \frac{1}{2h}I & \ddots & \ddots & \\ & \ddots & \ddots & -\frac{1}{2h}I\\ & & \frac{1}{2h}I & O \end{array}\right],\\ L=\left[\begin{array}{cccc} L & \frac{1}{h^{2}}I & & \\ \frac{1}{h^{2}}I & \ddots & \ddots & \\ & \ddots & \ddots & \frac{1}{h^{2}}I\\ & & \frac{1}{h^{2}}I & L \end{array}\right]. \end{array}$$

The above discretized linear system (5) can then be written in the form of *𝕂* **U** = **F**, where

(8) $$\begin{array}{c} K=\left[\begin{array}{cccc} A_{1} & A_{2} & O & O\\ D_{x} & D_{y} & O & -\frac{1}{\rho }I\\ I-\frac{\mu \Delta t}{\rho }L & O & \frac{\Delta t}{\rho }D_{x} & -\frac{\mu \Delta t}{3\rho^{2}}D_{x}\\ O & I-\frac{\mu \Delta t}{\rho }L & \frac{\Delta t}{\rho }D_{y} & -\frac{\mu \Delta t}{3\rho^{2}}D_{y} \end{array}\right],\\ U=\left[\begin{array}{c} u^{\left(n+1\right)}\\ v^{\left(n+1\right)}\\ p^{\left(n+1\right)}\\ s^{\left(n+1\right)} \end{array}\right],\;\;F=\left[\begin{array}{c} c^{\left(n+1\right)}\\ 0\\ k_{1}^{\left(n\right)}\\ k_{2}^{\left(n\right)} \end{array}\right]. \end{array}$$

For notational simplicity, we drop the superscripts (*n*) and (*n* + 1). Then, in the right-hand side, **u**, **v**, **p**, **s**, **c**, **k**_1_, and **k**_2_ are the (*M* × *N*) × 1 vectors defined as follows:

(9) $$\begin{array}{c} u=\left[\begin{array}{c} u_{\left(1,1\right)}\\ \vdots \\ u_{\left(N,M\right)} \end{array}\right],\;\;v=\left[\begin{array}{c} v_{\left(1,1\right)}\\ \vdots \\ v_{\left(N,M\right)} \end{array}\right],\;\;p=\left[\begin{array}{c} p_{\left(1,1\right)}\\ \vdots \\ p_{\left(N,M\right)} \end{array}\right],\;\;s=\left[\begin{array}{c} s_{\left(1,1\right)}\\ \vdots \\ s_{\left(N,M\right)} \end{array}\right],\;\;c=\left[\begin{array}{c} c_{\left(1,1\right)}\\ \vdots \\ c_{\left(N,M\right)} \end{array}\right],\\ k_{1}=\left[\begin{array}{c} u_{\left(1,1\right)}-\Delta t\left(u_{\left(1,1\right)}\frac{u_{\left(2,1\right)}-u_{\left(0,1\right)}}{2h}+v_{\left(1,1\right)}\frac{u_{\left(1,2\right)}-u_{\left(1,0\right)}}{2h}\right)\\ \vdots \\ u_{\left(N,M\right)}-\Delta t\left(u_{\left(N,M\right)}\frac{u_{\left(N+1,M\right)}-u_{\left(N-1,M\right)}}{2h}+v_{\left(N,M\right)}\frac{u_{\left(N,M+1\right)}-u_{\left(N,M-1\right)}}{2h}\right) \end{array}\right],\\ k_{2}=\left[\begin{array}{c} v_{\left(1,1\right)}-\Delta t\left(u_{\left(1,1\right)}\frac{v_{\left(2,1\right)}-v_{\left(0,1\right)}}{2h}+v_{\left(1,1\right)}\frac{v_{\left(1,2\right)}-v_{\left(1,0\right)}}{2h}\right)\\ \vdots \\ v_{\left(N,M\right)}-\Delta t\left(u_{\left(N,M\right)}\frac{v_{\left(N+1,M\right)}-v_{\left(N-1,M\right)}}{2h}+v_{\left(N,M\right)}\frac{v_{\left(N,M+1\right)}-v_{\left(N,M-1\right)}}{2h}\right) \end{array}\right]. \end{array}$$

However, the coefficient matrix in (8) is nonsymmetric, sparse, and large scale. Moreover, it has a bad condition number and is almost singular. Hence, we use the least-squares approach with Tikhonov regularization to solve the minimization problem with a regularization term of the form:

(10) $$\begin{array}{c} \hat{U}=arg\underset{u}{min}{\left\|KU-F\right\|}^{2}+{\left\|\Gamma U\right\|}^{2}. \end{array}$$

By setting Γ = *λ𝕀*_(4×*N*×*M*)×(4×*N*×*M*)_, the minimization problem is written as

(11) $$\begin{array}{c} \hat{U}=arg\underset{u}{min}{\left\|KU-F\right\|}^{2}+\lambda^{2}{\left\|U\right\|}^{2}, \end{array}$$

and its solution is given by

(12) $$\begin{array}{c} \hat{U}={\left(K^{T}K+\lambda^{2}I\right)}^{-1}K^{T}F. \end{array}$$

We perform the reconstruction algorithm using the heuristically selected Tikhonov regularization parameter *λ* shown in the reconstruction algorithm (11).

### 2.3. Numerical Simulations with Moving LV Boundaries

**Figure 3:**
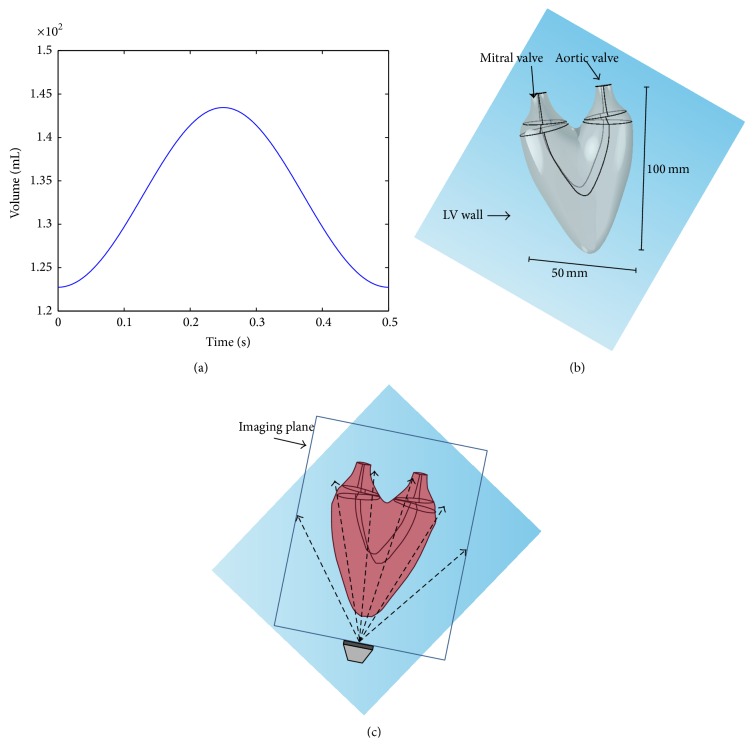
LV model for numerical experiments. (a) LV volume curve according to diastolic and systolic motions, (b) 3D LV shape model at the end systole, and (c) 2D imaging plane of A3CH view positioned in the constructed 3D LV model. The dashed arrows means the scanning lines transmitted from a linear array transducer for 2D imaging.

We obtain synthetic flow data inside a virtual moving simplified LV wall. To obtain the synthetic intraventricular flows, we construct a 3D moving LV region of the stroke volume of about 20 mL. As shown in Figure 3(a), the time-dependent LV volume ranges from 123 mL to 143 mL during the entire cycle of 0.5 s.  Figure 3(b) shows a LV shape model corresponding to the volume of 133 mL at the preset pressure state. We then perform a numerical simulation of the forward problem of the Navier-Stokes equation inside the 3D moving LV for the beat cycle. For the forward simulations, the “fluid-structure interaction model” of the COMSOL software is used, and 3D intraventricular velocity fields are computed as a solution of the forward problem. A no-slip boundary condition is used for computing the velocity fields. We assume that the blood flow during the filling of the LV is ejected in the normal direction to the inlet valve surface Γ^*I*^(*t*), that the flow velocity is uniform on the entire inlet boundary, and that the LV volume change is equal to the total amount of its net inflow:

(13) $$\begin{array}{c} \frac{\partial V\left(\Omega \left(t\right)\right)}{\partial t}=\int_{\Gamma^{I}\left(t\right)}n\cdot udS, \end{array}$$

where **n** is the normal vector to the mitral valve (inlet valve) boundary denoted by Γ^*I*^(*t*). The boundary portion containing the mitral and aortic valves was fixed not to be moved. Neumann boundary conditions of velocity fields were given by setting the zero normal derivatives at the outlet Γ^*O*^ and inlet Γ^*I*^ valves for the LV diastole and systole, respectively. For simplicity, pressure is assumed to be a constant at the inlet, while the Neumann condition for the pressure is used at the outlet. (The work of [16, 17] will also help readers create synthetic flow data.)

**Figure 4:**
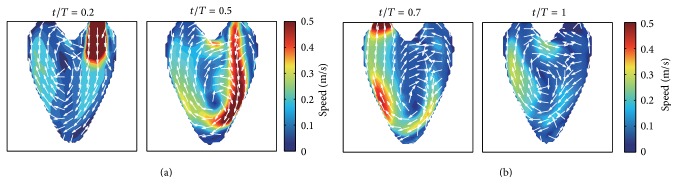
Synthetic intraventricular velocity fields projected on the imaging plane. (a) and (b) show the velocity fields for the diastole and systole process of LV, respectively.

We project the 3D synthetic velocity fields on the imaging plane of A3CH view, which is set to be located in the constructed 3D LV model (see Figure 3(c)). The projected 2D velocity fields are used as reference data to evaluate the proposed 2D velocity reconstruction algorithm. Representative cases of these synthetic 2D flows are depicted in Figure 4, which show the time-varying dominant vortex patterns inside the virtual moving LV wall. While the LV model is relaxing, flows enter into the LV through the inlet valve. The main stream impinges against the LV wall, and a large vortex is simultaneously formed near the center. The large vortex moves upward during the contraction process and weakens at the end of the systole cycle.

**Figure 5:**
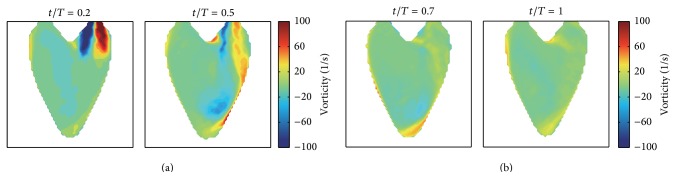
Vorticities corresponding to the synthetic 2D intraventricular velocity fields. (a) and (b) show the vorticity changes according to the diastole and systole process of LV, respectively.

Figure 5 shows the vorticity fields obtained by taking the curl operator to the synthetic flow fields. At the early stage of relaxation, small vortices are formed on the both sides of the main incoming stream. The higher vorticity near the wall is related to the fact that the high momentum incoming flow results in increasing the wall shear stress when the incoming flow impinges to the wall; we think. While the vorticity gradually grows during the expanding process, it eventually shrinks and weakens during the process of contraction.

**Figure 6:**
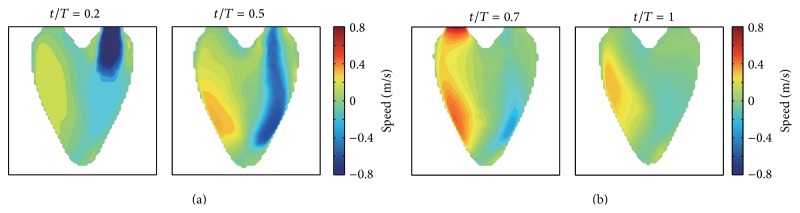
One-directional velocity component data. (a) and (b) show the scanline directional components of the generated velocity fields during the diastole and systole process of LV, respectively.

The inner product of the synthetic 2D velocity fields and the scanline directional unit vectors gives the scanline directional velocity components, which can be regarded as the color flow data of the intraventricular flow on the A3CH view measured by a real ultrasound system. The one-directional velocity component data are used as the input data for our proposed method.  Figure 6 illustrates the scanline directional velocity components. The figures in the second column corresponding to the end-diastole and end-systole reflect the overall similar flow patterns that are comparable to real color patterns (see Figures 12(c) and 12(d) in Appendix B).

### 2.4. Choice of the Parameter ***λ***

**Figure 7:**
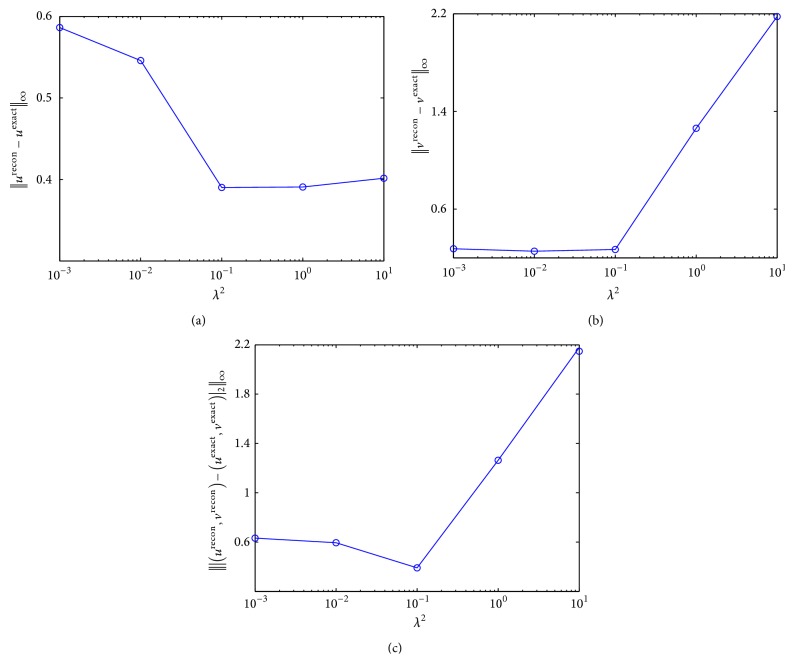
Error change depending on the parameter *λ*. (a) *L*_*∞*_-norm errors between *u* ^recon^ and *u* ^exact^, (b) between *v* ^recon^ and *v* ^exact^, and (c) between (*u* ^recon^, *v* ^recon^) and (*u* ^exact^, *v* ^exact^) with respect to *λ* ^2^. Here, (*u* ^recon^, *v* ^recon^) and (*u* ^exact^, *v* ^exact^) stand for the reconstructed and the reference velocity fields, respectively.

In this study, all the experiments were performed by setting the parameter $\lambda =\sqrt{0.1}$. To optimize the value of this parameter, we investigated the errors of the reconstructed velocity fields with respect to the change of *λ* ^2^. We adjusted the value of *λ* ^2^ to each power of 10 from 10^−3^ to 10^1^.  Figure 7 shows that the *L*_*∞*_-norm errors between the reconstructed velocity fields and the reference data have formed a convex plot with the minimized *L*_*∞*_-norm error attained at *λ* ^2^ = 0.1.

## 3. Results

**Figure 8:**
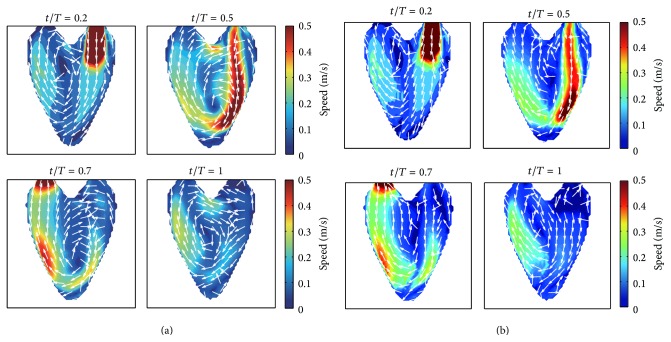
Reconstruction results. The reference vector field (a) and the reconstructed 2D vector fields by the proposed method (b).

We demonstrated the feasibility of the proposed method by showing the *L*_2_-norm errors between the synthetic flow and the reconstructed flow fields. We reconstructed the velocity fields from the synthetic scanline directional velocity components by repeatedly solving the minimization problem (11) five times for the entire beat cycle.  Figure 8 shows the reconstruction results of velocity fields obtained from the input data of the one-directional velocity components. As in the forward numerical simulations, the incoming stream impinged to the lower right wall. Overall, the reconstructed flows showed very similar patterns to the reference flows shown in Figure 4. However, the main vortex near the center was shifted upward relative to the reference vector field. This shifting phenomenon was also continued in the remaining steps.

**Figure 9:**
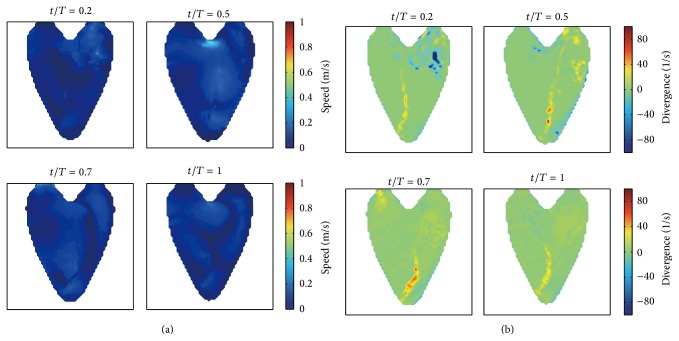
Errors of the reconstruction results. (a) 2D plots for error-magnitude of reconstructed (*u*, *v*) relative to the reference velocity fields and (b) the mass source term *s*.

Figure 9 illustrates the performance of the proposed model. Part (a) compares the pointwise error magnitude images of the reconstructed velocity fields with the reference data. While the reconstructed *u*-component errors were distributed in the range of  −0.39~0.25 m/s, the errors of the *v*-components showed larger differences of  −0.24~0.27 m/s for the entire cycle.  Figure 9(b) shows the distribution of the mass source term *s* and reflects the out-of-plane components of the flows. The ratio *s*/*ρ* ranged from −151.1 s^−1^ to 82.5 s^−1^. From these contributions of *s*, the averaged pointwise errors for *u*- and *v*-components were 0.06 m/s and 0.02 m/s, respectively. The averaged pointwise error-magnitude was 0.065 m/s. We also compared the reconstruction results between the 2D Navier-Stokes models with and without the mass source term *s*. When *s* was used, the largest pointwise errors for the *u*- and *v*-components were 0.39 m/s and 0.27 m/s, respectively; reconstruction results not considering *s* showed larger pointwise errors of 0.71 m/s and 0.78 m/s for these components, respectively, for the entire cycle. These results support that the proposed model improves reconstruction performance.

For further quantitative comparison, we computed *L*_2_-norm errors of each velocity field on the given whole region at each 1/10 time step of the whole cycle.  Table 1 shows the relatively large velocity error (≈47%) for the end of the contraction process compared with the expanding process. While the pointwise errors of velocity may be larger than its *L*_2_-norm errors at some local regions, those pointwise errors at the local regions did not affect the major features of the vortex flows. This implies that the proposed model is reasonably accurate for estimating the unsteady features of the blood flows when the reconstruction algorithm of flow is performed repeatedly over several cycles of diastole and systole processes.

**Figure 10:**
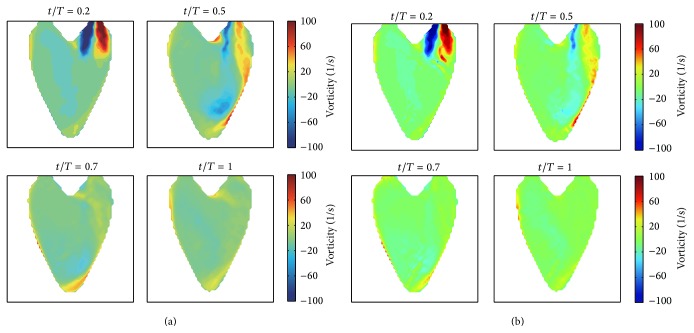
Reconstruction results of vorticities. These were obtained by taking the 2D curl operator to the reconstructed velocity fields.

**Figure 12:**
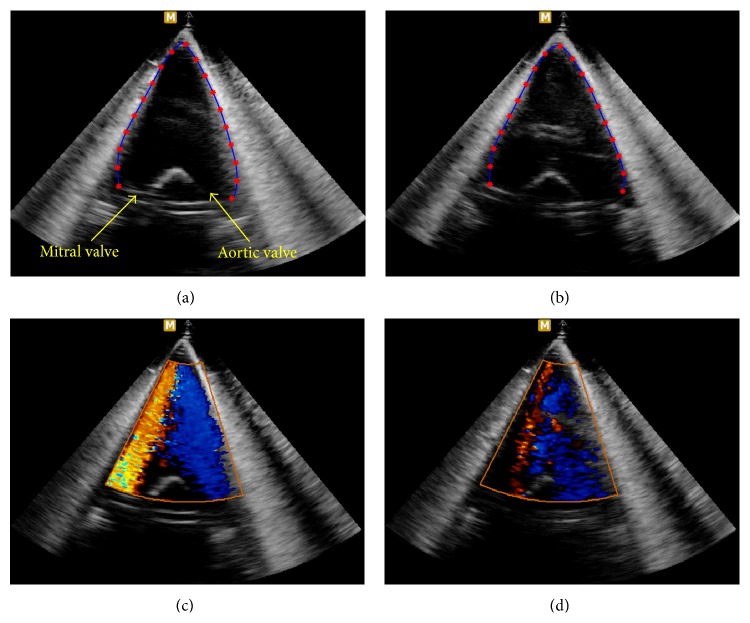
Real data acquisition using LV phantom and border extraction. (a) and (b) are echo images in ventricular shape at end-diastole and end-systole phases, respectively. (c) and (d) are color flow images corresponding to (a) and (b), respectively.

Although the proposed reconstruction method produced nonsmooth distributions of vorticity inside the LV region (as shown in Figure 10), the evolutions of the large vortical structure inside the LV region were clearly observed, and the vorticities overall showed very similar patterns to the reference results described in Figure 5. The pointwise and global errors for the vortex fields are listed in Table 2. These nonsmooth vorticity distributions may be induced by uncertainties of the boundary geometry and the regularization term in the minimization problem (11).

## 4. Discussion and Conclusions

We propose here a new 2D Navier-Stokes model that reconstructs intraventricular flows using color flow data and LV boundaries extracted from echocardiography data. The proposed model considers both in-plane and out-of-plane blood flows on the imaging plane of an apical long-axis three-chamber view. The out-of-plane components moving out of the imaging plane were modeled as the mass source term of a source-sink distribution. We reconstructed blood flows in the imaging domain by solving a system of equations, which includes the 2D incompressible Navier-Stokes equation of the mass source term and a color flow measurement equation describing the one-directional velocity component of the color flow data. The boundary conditions that are required to solve the system of equations are given by the LV borders extracted from echocardiography data. To evaluate the proposed method, numerical experiments were performed on synthetic flow data following a virtual LV motion. The results showed that the proposed method is feasible and potentially valuable for reconstructing intracardiac flow fields.

The numerical experiments used an imaging domain of size 64 × 64 pixels, because the computational costs of the proposed reconstruction method were large. We divided the whole cycle into 1000 time-steps for stable numerical implementation and repeatedly performed the proposed algorithm five times for the entire heartbeat cycle. We observed that the solutions obtained after two repeats were very similar to each other. A detailed mathematical description of the convergence of the solution will be given in our next work.

All the experiments were conducted using MATLAB 7.10.0, and the computational time for each step was about 12 s using an Intel i7-4702MQ Quadcore CPU running at 2.0 GHz with 8 GB of RAM. The speed of the proposed method needs to be improved for its practical application, and a study to reduce the processing time is under way.

To reconstruct 2D flow patterns using the color flow images requires an experimental ultrasound system that can acquire echo and color data independently; however, we had only a commercial ultrasound system that cannot support data acquisition for the experiments. The frame-rate limitation of the color flow data acquired by the ultrasound imaging system should also be overcome. The color flow images from the ultrasound imaging system are obtained by applying the autocorrelation algorithm [13] after repeatedly transmitting and receiving the ultrasound beam along the same scanline; this then causes the low frame-rate of color flow data acquisition. The proposed method assumed that the time difference between the sequential color flow images is very small and that each color value reflects the exact velocity components in the direction of ultrasound beam propagation. For the proposed method to be practically applicable, the frame-rate needs to be improved. The development of an ultrafast imaging system is ongoing. Such a system is expected to have a frame-rate higher than 1000 fps using plane wave beam-forming; it would acquire echo and color flow image data separately and support the performance of the proposed method in real application. Some recent works by other researchers have examined ultrafast flow imaging [18–20]. A review of these references will help us to perform our future work on an ultrafast imaging system. In Appendix B, we describe an* in vitro* phantom experimental setup for empirical data (color flow ultrasound and LV boundary data) to demonstrate the feasibility of our reconstruction method.

To advance the proposed algorithm, we are studying on mitral valve tracking. Based on reports [21, 22] that the motion of the mitral valve affects the vorticities of the intraventricular flows, studies on the boundary conditions containing the valve motion are under way.

This work is the first that describes overall our proposed method. More detailed mathematical analysis and further validation tests may be required to verify its scientific validity and practical applicability. We will perform several follow-up works to achieve this.

## Figures and Tables

**Figure 11:**
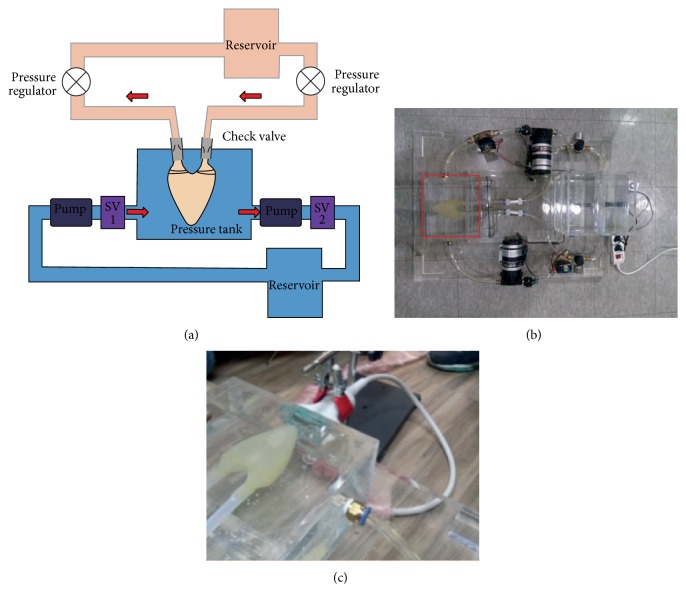
LV phantom operating system. The LV phantom is made of polyurethane. We control the fluid motion by the synchronous operation of two pumps.

**Figure 13:**
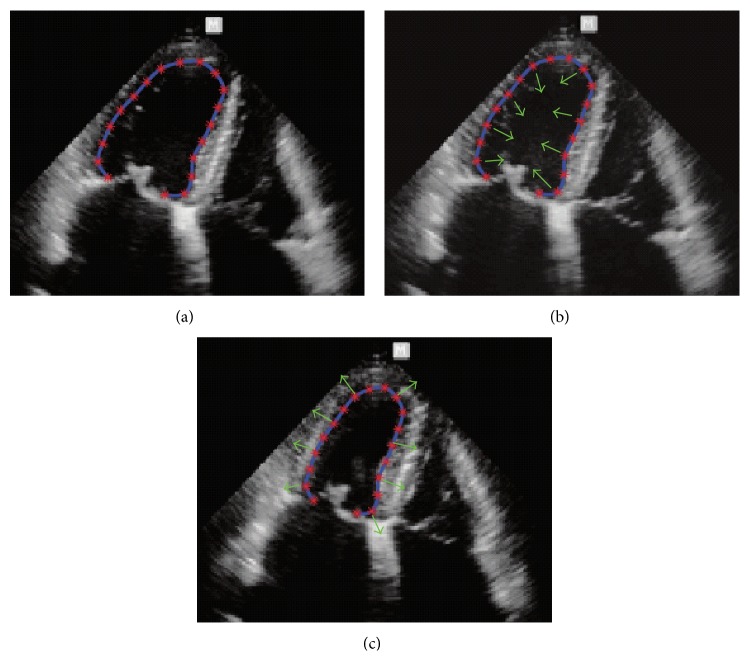
Velocities of wall motion using LV border tracking method. Initialized LV contour at end-diastole frame (a), velocity fields of contracting and expanding LV wall (b-c). Blue solid line, red dots, and green arrows are the LV contours *𝒞*(*t*), contour points {**x**_1_(*t*),…, **x**_*n*_(*t*)}, and some of velocity vectors {**v**_1_(*t*),…, **v**_*n*_(*t*)}, respectively.

**Table 1:** Comparison of *L*_2_-norm errors for the velocity fields recovered from the synthetic and real data. The third and fourth columns represent normalized *L*_2_-norm errors for pointwise and global energy estimates of velocity. Here, ${\left\|v\right\|}_{2}=\sqrt{u^{2}+v^{2}}$, Δ**v** = **v** ^recon^ − **v** ^exact^, and Δ*k*/*k* ^exact^ = |‖**v** ^exact^‖_2_ − ‖**v** ^recon^‖_2_|/‖**v** ^exact^‖_2_.

*t*/*T*	‖**v** ^exact^‖_2_	‖**v** ^recon^‖_2_	‖Δ**v**‖_2_/‖**v** ^exact^‖_2_	Δ*k*/*k* ^exact^
0.1	0.012	0.011	28.2%	11.1%
0.2	0.026	0.023	17.6%	8.9%
0.3	0.033	0.029	21.2%	9.9%
0.4	0.028	0.024	28.2%	12.0%
0.5	0.018	0.015	39.6%	17.4%
0.6	0.015	0.013	39.8%	16.8%
0.7	0.015	0.013	36.6%	13.4%
0.8	0.014	0.012	36.3%	12.0%
0.9	0.011	0.010	39.3%	13.0%
1.0	0.010	0.007	47.5%	18.6%

**Table 2:** Comparison of *L*_2_-norm errors for the corresponding vortex fields. The third and fourth columns represent normalized *L*_2_-norm errors for pointwise and global energy estimates of vorticity. Here, Δ*ω* = *ω* ^recon^ − *ω* ^exact^ and Δ*k*_*ω*_/*k*_*ω*_^exact^ = |‖*ω* ^exact^‖_2_ − ‖*ω* ^recon^‖_2_|/‖*ω* ^exact^‖_2_.

*t*/*T*	‖*ω* ^exact^‖_2_	‖*ω* ^recon^‖_2_	‖Δ*ω*‖_2_/‖*ω* ^exact^‖_2_	Δ*k*_*ω*_/*k*_*ω*_^exact^

0.1	1.09	1.19	36.8%	8.7%
0.2	2.35	2.48	24.7%	5.3%
0.3	2.82	2.90	21.7%	2.8%
0.4	2.31	2.34	21.2%	1.4%
0.5	1.67	1.67	32.1%	0.4%
0.6	1.38	1.40	40.6%	1.7%
0.7	1.33	1.40	46.7%	5.2%
0.8	1.20	1.30	51.4%	7.8%
0.9	0.95	1.02	52.0%	6.7%
1.0	0.79	0.82	47.8%	4.2%

## Appendices

### A. Mathematical Formulation

Based on the 3D modeling of intraventricular blood flows, we derive a 2D mathematical model describing the relationship between blood flows and color flow data measured on the imaging plane.

#### A.1. Mathematical Model and Inverse Problem

Let *D* be a 3D imaging domain, *Ω*(*t*) a time-varying LV region satisfying *Ω*(*t*)⊆*D*⊆*ℝ* ^3^, and *T* a beat cycle. For the beat cycle *T*, we consider a spatial-temporal domain *Ω*_*T*_ defined by *Ω*_*T*_ : = ⋃_0<*t*<*T*_ *Ω*(*t*)×{*t*}⊆*D* × (0, *T*). Let **v**(**r**, *t*) be a velocity field of the blood flow within the spatial-temporal domain *Ω*_*T*_, **a**(**r**) = (*a*_1_(**r**), *a*_2_(**r**), *a*_3_(**r**)) scanline directional unit vectors at the position **r** ∈ *D* and *c*(**r**, *t*) color flow data in *Ω*_*T*_. Given *c*(**r**, *t*), we then consider an inverse problem to find a 3D vector field **v** = (*u*, *v*, *w*) satisfying the following condition:

(A.1) $$\begin{array}{c} a\left(r\right)\cdot v\left(r,t\right)=c\left(r,t\right), \end{array}$$

where · is the inner product operator.

To solve this inverse problem, we should deal with the 3D Navier-Stokes equations governing the blood flow **v**:

(A.2) $$\begin{array}{c} \rho \left(\frac{\partial v}{\partial t}+v\cdot \nabla v\right)=-\nabla p+\mu \nabla^{2}v\;in\;\;\Omega_{T},\\ \nabla \cdot v=0\;in\;\;\Omega_{T}. \end{array}$$

However, given that our aim is to reconstruct the blood flow using 2D measurements, we reduce (A.1) and (A.2) to a 2D inverse problem and the corresponding 2D Navier-Stokes equation. We compute the velocity fields of blood flows by using the scanline directional projected velocity components represented as 2D color flow data in a commonly used ultrasound 2D imaging scanner and then solving the system of equations related to them.

#### A.2. Mathematical Model on a 2D Imaging Plane

From now on, *Ω*(*t*) denotes the cross-section of the LV region in the A3CH view. (For simplicity, the same notation *Ω*(*t*) in 3D will be used to represent its cross-section.) The notations of Γ^*I*^(*t*) and Γ^*O*^(*t*) are same as shown in Figure 2. Let *D* be a 2D imaging domain satisfying *Ω*(*t*)⊆*D*⊆*ℝ* ^2^ so that it is equal to the cross-sectioned region of the 3D imaging domain by the A3CH view. As described earlier, we try to model flows in the 2D imaging plane *D*, where color flow data are practically measured. In this case, the scanline direction **a**(**r**) is given to be tangent to the plane *D*,  *a*_3_ = 0 on the plane. Let *c*(**x**, *t*) be the measured color flow data at the position **x** ∈ *D* at time *t*. The color flow data *c*(**x**, *t*) can then be expressed as the inner product of the scanline vector and the velocity vector:

(A.3) $$\begin{array}{c} c\left(x,t\right)=\left(a_{1}\left(x\right),a_{2}\left(x\right)\right)\cdot \left(u\left(x,t\right),v\left(x,t\right)\right). \end{array}$$

Hence, the corresponding inverse problem is to recover (*u*, *v*) from knowledge of *c* on the imaging plane *D*. Also, (A.2) can be rewritten as

(A.4) ∂u∂t+u∂u∂x+v∂u∂y=−1ρ∂p∂x+μρ∂2u∂x2+∂2u∂y2+μρ∂2u∂z2−w∂u∂z︸f1,∂v∂t+u∂v∂x+v∂v∂y=−1ρ∂p∂y+μρ∂2v∂x2+∂2v∂y2+μρ∂2v∂z2−w∂v∂z︸f2,∂u∂x+∂v∂y=−∂w∂z.

Note that color flow data may not contain measurable information on the 2D plane *D* for *f*_1_ = (*μ*/*ρ*)(∂^2^ *u*/∂*z* ^2^) − *w*(∂*u*/∂*z*), *f*_2_ = (*μ*/*ρ*)(∂^2^ *v*/∂*z* ^2^) − *w*(∂*v*/∂*z*), and ∂*w*/∂*z*. We want to keep the incompressible condition of the 3D problem. However, the third term ∂*w*/∂*z* of the divergence of 3D flow is neither of the measurable or computable quantities in the 2D plane. Here, we model it as a mass source-sink term *s*, which represents the linear deformation of the fluid in the out-of-plane direction. This new unknown term will be determined by solving the modified Navier-Stokes equation with the color flow measurement data. Introducing a new variable *s*, we obtain the following reduced 2D Navier-Stokes model:

(A.5) $$\begin{array}{c} \frac{\partial u}{\partial t}+u\frac{\partial u}{\partial x}+v\frac{\partial u}{\partial y}=-\frac{1}{\rho }\frac{\partial p}{\partial x}+\frac{\mu }{\rho }\nabla^{2}u+\frac{\mu }{3\rho^{2}}\frac{\partial s}{\partial x},\\ \frac{\partial v}{\partial t}+u\frac{\partial v}{\partial x}+v\frac{\partial v}{\partial y}=-\frac{1}{\rho }\frac{\partial p}{\partial y}+\frac{\mu }{\rho }\nabla^{2}v+\frac{\mu }{3\rho^{2}}\frac{\partial s}{\partial y},\\ \frac{\partial u}{\partial x}+\frac{\partial v}{\partial y}=\frac{s}{\rho }. \end{array}$$

This model is equivalent to a 2D incompressible flow with a source-sink distribution *s*(**x**, *t*) [15]. In fact, if we do not consider the external force, equation (3.3.13) in [15] implies

(A.6) ρ∂u∂t+u∂u∂x+v∂u∂y=−∂p∂x+μ∇2u+μ3ρ∂∂x∂u∂x+∂v∂y,ρ∂v∂t+u∂v∂x+v∂v∂y=−∂p∂y+μ∇2v+μ3ρ∂∂y∂u∂x+∂v∂y.

### B. *In Vitro* Phantom Experiments

#### B.1. Experimental Setup

The experimental setup for operating the LV phantom is depicted in Figure 11. The setup was composed of main two parts: one for making the LV phantom beat periodically and the circulatory part (resp., the lower and upper parts in Figure 11(a)). The fluidic motion was controlled by the synchronous operation of two pumps, two solenoid valves (SVs), and the check valves employed to model the aortic and mitral valves. The phantom, which was constructed of polyurethane, was immersed in a water tank. The phantom was 3D printed as a half-ellipsoid [23, 24]. The experimental circulatory system including the LV phantom was completely filled with water. Figures 11(b) and 11(c) show the manufactured LV phantom with its design drawing and the experimental setting for scanning the LV phantom, respectively. For LV systolic motion, the left solenoid valve (SV1) is set to open, while the right solenoid valve (SV2) is closed. The pressure inside the tank then increases, which subsequently exerts pressure onto the LV phantom. When the pressure inside the LV phantom is greater than the preset pressure of the check valve, the fluid in the LV phantom surges into the circulatory system. The check valves restrain backward flow, making the fluid in the LV phantom pass through the aortic valve to circulate the system. Dilation of the LV phantom then follows by closing SV1 and opening SV2, allowing similar complementary processes to occur. A simple timer switch controls the opening and closing of the solenoid valves to mimic LV beating. The volume of the LV phantom under the preset pressure was measured to be 133 mL, while the stroke volume was about 20 mL during LV beating with 0.5 s period. The polyurethane is flexible and inelastic; the LV phantom therefore may retain a constant “endocardial” surface area, while the contained volume may vary owing to changes in its cross-sectional shape. Despite those limitations, vortex flow pattern inside the phantom is generated during the “diastole process.” The B-mode and C-mode images were scanned using an Accuvix V10 ultrasound system (Samsung Medison, Seoul, South Korea) and a probe P2-4BA with the frequency band of 2~4 MHz. The probe was positioned just below the water surface, at a depth of approximately 10 cm, to enable the LV phantom to be positioned in the center of the ultrasound image.

Representative ultrasound echo and color flow images acquired by scanning the LV phantom are shown in the first and second rows of Figure 12, respectively. By applying an LV tracking algorithm to the acquired ultrasound echo images, we obtained LV boundaries for the whole cycle. (The LV tracking method is explained in the next subsection.) The red dotted and blue lines in Figures 12(a) and 12(b) present the LV boundaries extracted in the end-diastole and end-systole images. The LV boundaries are extracted at each frame (50 fps) during the cardiac cycle in order to impose moving boundary conditions for the numerical simulations of the next section. Figures 12(c) and 12(d) show that the color flow patterns may indicate two different vortex flows. Here, the red and blue colors represent the velocity components of the flows coming toward and receding from the ultrasound probe, respectively. The color flow pattern in the diastolic phase is clearly stronger than that in the systolic phase, as has been reported for human LV [1].

#### B.2. LV Wall Segmentation from B-Mode Image

We acquire ultrasound echo images for the entire cardiac cycle and from them extract the LV borders, typically by myocardial motion tracking. The myocardial motion tracking method is combined with the Lucas-Kanade method and a constraint formulated by the global deformation of nonrigid heart motion proposed in [25]. As illustrated in Figure 13, we denote the endocardial border traced at initially selected end-diastole frame by a parametric contour *𝒞* ^*∗*^ = {**x** ^*∗*^(*s*) = (*x* ^*∗*^(*s*), *y* ^*∗*^(*s*))∣0 ≤ *s* ≤ 1} that can be identified as its *n* tracking points **x**_1_^*∗*^ = **r** ^*∗*^(*s*_1_),…, **x**_*n*_^*∗*^ = **x** ^*∗*^(*s*_*n*_). Here, 0 = *s*_1_ < *s*_2_ < ⋯<*s*_*n*_ = 1. Let *𝒞*(*t*) = {**x**(*s*, *t*) = (*x*(*s*, *t*), *y*(*s*, *t*))∣0 ≤ *s* ≤ 1} be the contour deformed from *𝒞*(0) = *𝒞* ^*∗*^ at time *t*. The velocity field **V**(*t*) of motion of the contour *𝒞*(*t*) is determined by a time change of tracking points {**x**_1_(*t*),…, **x**_*n*_(*t*)}:

(B.1) $$\begin{array}{c} V\left(t\right):=\left[\begin{array}{c} v_{1}\left(t\right)\\ \vdots \\ v_{n}\left(t\right) \end{array}\right]=\frac{d}{dt}\left[\begin{array}{c} x_{1}\left(t\right)\\ \vdots \\ x_{n}\left(t\right) \end{array}\right]\\ with\;\;\left[\begin{array}{c} x_{1}\left(0\right)\\ \vdots \\ x_{n}\left(0\right) \end{array}\right]=\left[\begin{array}{c} x_{1}^{*}\\ \vdots \\ x_{n}^{*} \end{array}\right]. \end{array}$$

Here, we identify the contour *𝒞*(*t*) with tracking points {**x**_1_(*t*),…, **x**_*n*_(*t*)}.
